# moxMaple3: a Photoswitchable Fluorescent Protein for PALM and Protein Highlighting in Oxidizing Cellular Environments

**DOI:** 10.1038/s41598-018-32955-5

**Published:** 2018-10-03

**Authors:** Andrii A. Kaberniuk, Manuel A. Mohr, Vladislav V. Verkhusha, Erik Lee Snapp

**Affiliations:** 1 Department of Anatomy and Structural Biology and Gruss-Lipper Biophotonics Center, Albert Einstein College of Medicine, 1300 Morris Park Avenue, Bronx, NY 10461 USA; 2 Janelia Research Campus, HHMI, 19700 Helix Drive, Ashburn, VA 20147 USA

## Abstract

The ability of fluorescent proteins (FPs) to fold robustly is fundamental to the autocatalytic formation of the chromophore. While the importance of the tertiary protein structure is well appreciated, the impact of individual amino acid mutations for FPs is often not intuitive and requires direct testing. In this study, we describe the engineering of a monomeric photoswitchable FP, moxMaple3, for use in oxidizing cellular environments, especially the eukaryotic secretory pathway. Surprisingly, a point mutation to replace a cysteine substantially improved the yield of correctly folded FP capable of chromophore formation, regardless of cellular environment. The improved folding of moxMaple3 increases the fraction of visibly tagged fusion proteins, as well as FP performance in PALM super-resolution microscopy, and thus makes moxMaple3 a robust monomeric FP choice for PALM and optical highlighting applications.

## Introduction

While the utility of fluorescent proteins (FPs) has revolutionized cell biology^1,2^, there has been a need for inert environmentally insensitive FPs. This is because different cellular environments can impair FP performance. For example, FPs can exhibit decreased fluorescence at lower pH values or in response to some ions, fold poorly at higher temperatures or can be inappropriately post-translationally modified in some cellular compartments^3–8^. The evolution and engineering of cytoplasmic FPs in the relatively neutral cytoplasm of organisms, such as jellyfish and bacteria, has produced FPs that often lack suitability for the many cellular environments that researchers study^6^. In particular, secretory proteins in eukaryotic cells can undergo N-linked glycosylation on consensus peptides of N-X-S/T (where X is any amino acid except proline), cysteines can form inter or intra-chain disulphide bonds not present in the cytoplasm, and the different compartments of the secretory pathway range in pH from neutral in the endoplasmic reticulum (ER) to a very acidic 4.0 in the lysosome^6^. Consequently, these post-translational modifications and environmental effects can lead to FP misfolding, dark FPs, and mislocalization^3–6^. Recently, our groups successfully engineered FPs into “mox” (monomeric nonoxidizing or disulphide bonding) forms to improve their suitability for a wide variety of eukaryotic cellular compartments^5,9^. Here we turn our attention to develop an improved photoswitchable FP to optically highlight fusion proteins to study their fate and for single-molecule-based photoactivation localization microscopy PALM super-resolution imaging^10–12^.

**Figure 1:**
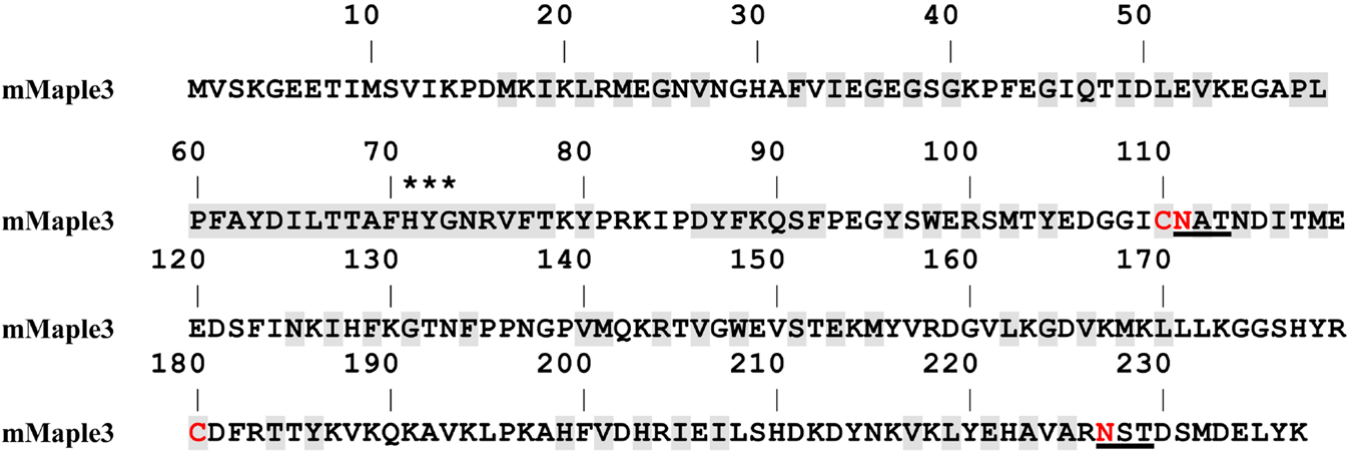
Amino acids sequence of mMaple3. Residues buried inside of the FP’s β-barrels are shaded, asparagine glycosylation tripeptides are underlined, and cysteines and asparagines to be mutated are colored red. Asterisks indicate the residues that form the chromophore.

The starting FP for this effort was required to satisfy several constraints. First, we focused on photoswitchable FPs (PSFPs) that have been optimized for single-molecule-based super-resolution imaging^12^. In particular, such FPs have high numbers of photons emitted per switching cycle, which impacts the localization precision of individual molecules; ii) a small ratio for the on- and off-switching rate constant, which limits the achievable localization density, and iii) a high signaling efficiency determining the fraction of target–PSFP fusion proteins detectable in a cell^13^. Due to the importance of avoiding unwanted protein- protein interactions^14,15^, the FP should be robustly monomeric. The recently described *Clavularia* coral-derived green-to-red photoswitching mMaple3 met all of these criteria and became the starting FP for our efforts^13^. Interestingly, mTFP1, the cyan parent FP from *Clavularia*^16^, is a rare example in the literature of an FP that enters and traffics through the eukaryotic secretory pathway. mTFP1 has a natural N-linked glycosylation consensus sequence near the COOH terminus of the protein and no cysteines. However, the engineering and evolution of mMaple3 incorporated several additional undesirable mutations including two cysteines and a N-linked glycosylation consensus sequence (Fig. 1). We sought to modify mMaple3 for use in oxidizing cellular environments.

## Results

### Evolution of mMaple3

Our engineering efforts began by modifying the coding sequence of mMaple3 using codons selected for high expression in mammalian cells – a strategy that was first used successfully in the development of EGFP^17^. Following synthesis of codon optimized mMaple3, we began to mutate selected amino acids.

The limiting step in mutagenesis of FPs for oxidizing environments is the replacement of cysteines with alternative amino acids. It was not obvious *a priori* that the cysteines of mMaple3 could be mutated without disrupting the desirable properties of mMaple3. Similar efforts for EGFP family members often resulted in dark proteins and our previous efforts with the development of moxEos3.2 and moxDendra2^9^ had revealed that the cysteine positions were highly sensitive even to seemingly conservative small amino acid substitutions. Serine was never tolerated and an alanine or valine could produce dramatic differences in apparent brightness^3,5,18^. As other related photoswitchable FPs mEos2, Dendra2, and mKikGR have cysteines at the identical positions^19^, we leveraged our previous successful efforts to engineer moxDendra2 whose cysteines align with those in mMaple3^20^.

**Figure 2:**
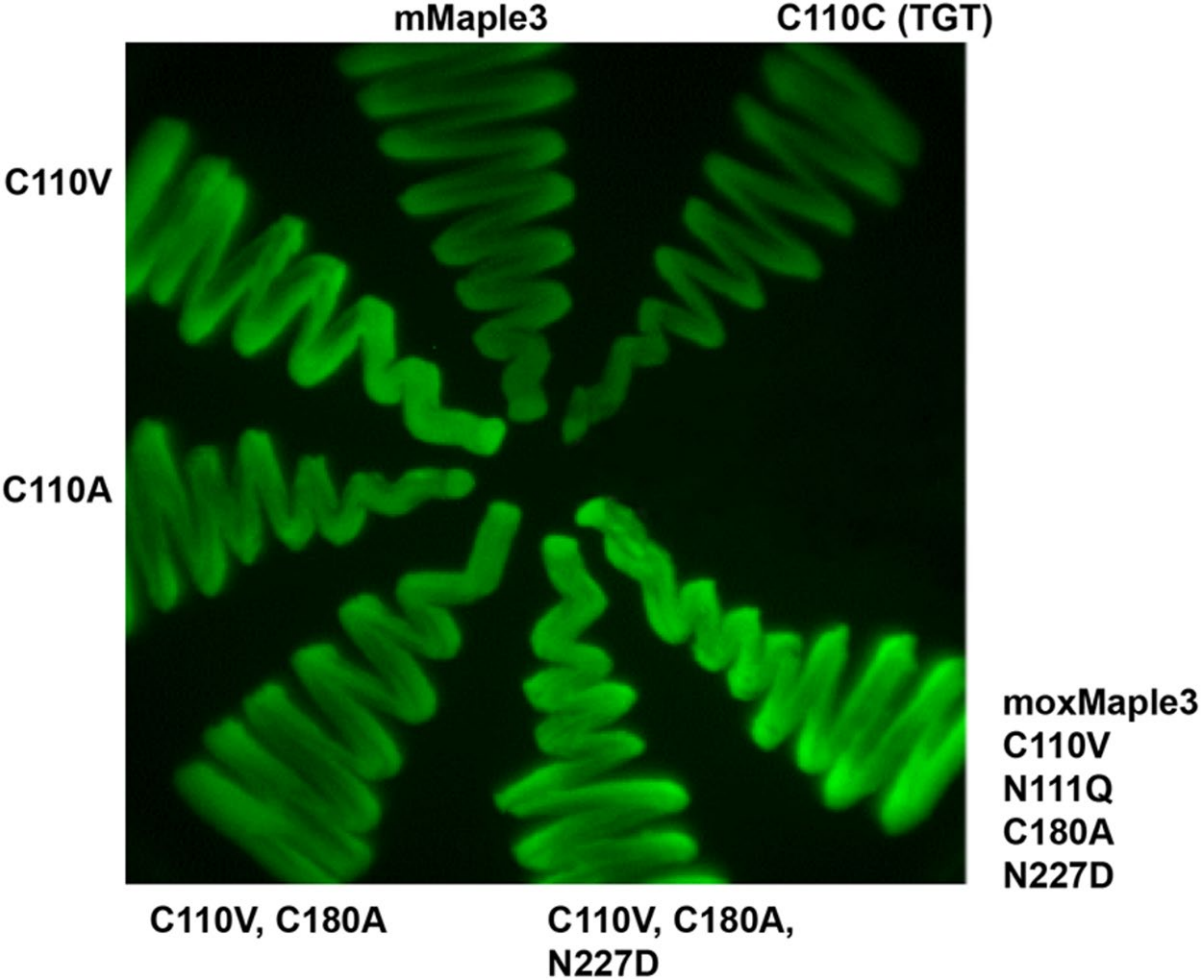
Mutagenesis improves FP signal. Streaks of bacterial strains induced to express the indicated mutant were grown on a Petri dish and then imaged using a stereomicroscope equipped with 480/40 nm excitation and 530/40 nm emission filters.

Initial screening of mutants was carried out by expressing the variants in bacteria and scoring bacterial streaks for whether they remained fluorescent. Using this standard approach, we were surprised by the results of the first cysteine mutants. Based on results with moxDendra2, we anticipated that Cys110 would maintain fluorescence if switched to an Ala or Val. While this hypothesis proved correct, we found that a Val mutation greatly increased the brightness of the bacteria (Fig. 2 and Table 1). A similar mutation of Cys180 did not increase fluorescence and a Val mutation at that position even decreased fluorescence, whereas an Ala mutation was tolerated with no impact on fluorescence. The cysteine mutations were combined and then glycosylation disrupting N to Q or D mutations were also made without any obvious impact on fluorescence (Fig. 2 and Table 1). Together, the mutations produced a sugarless, cysteine-free FP we term moxMaple3 (Fig. 2). Bacteria expressing the resulting protein were fluorescent and exhibited the increased brightness observed with the single C110V mutation.

**Table 1:** Brightness of bacteria expressing mMaple3 mutants.

Construct	Normalized Brightness
**mMaple3 wt**	1.00
C110C (TGT)	0.99, 1.06
C110V (GTC) (clone 1)	1.98, 1.98
C110V (GTC) (clone 2)	1.69, 1.76, 1.55
C110V (GTG)	1.74
C110V (GTT)	1.75
C110A (GCC)	1.49
C110A (GCG)	1.07, 1.04
C110I (ATC)	1.37, 1.12
C110I (ATA)	1.24
C110T (ACT)	0.37
C110V (GTC), N111Q (CAG)	1.94
C180A (GCC)	0.78
C180V (GTC)	0.67
N227D (GAC)	1.05
C110V (GTC), C180A (GCC)	1.61
C110V (GTC), C180A (GCC), N227D	1.79
**moxMaple3**	
C110V, N111Q, C180A, N227D	2.28

**Figure 3:**
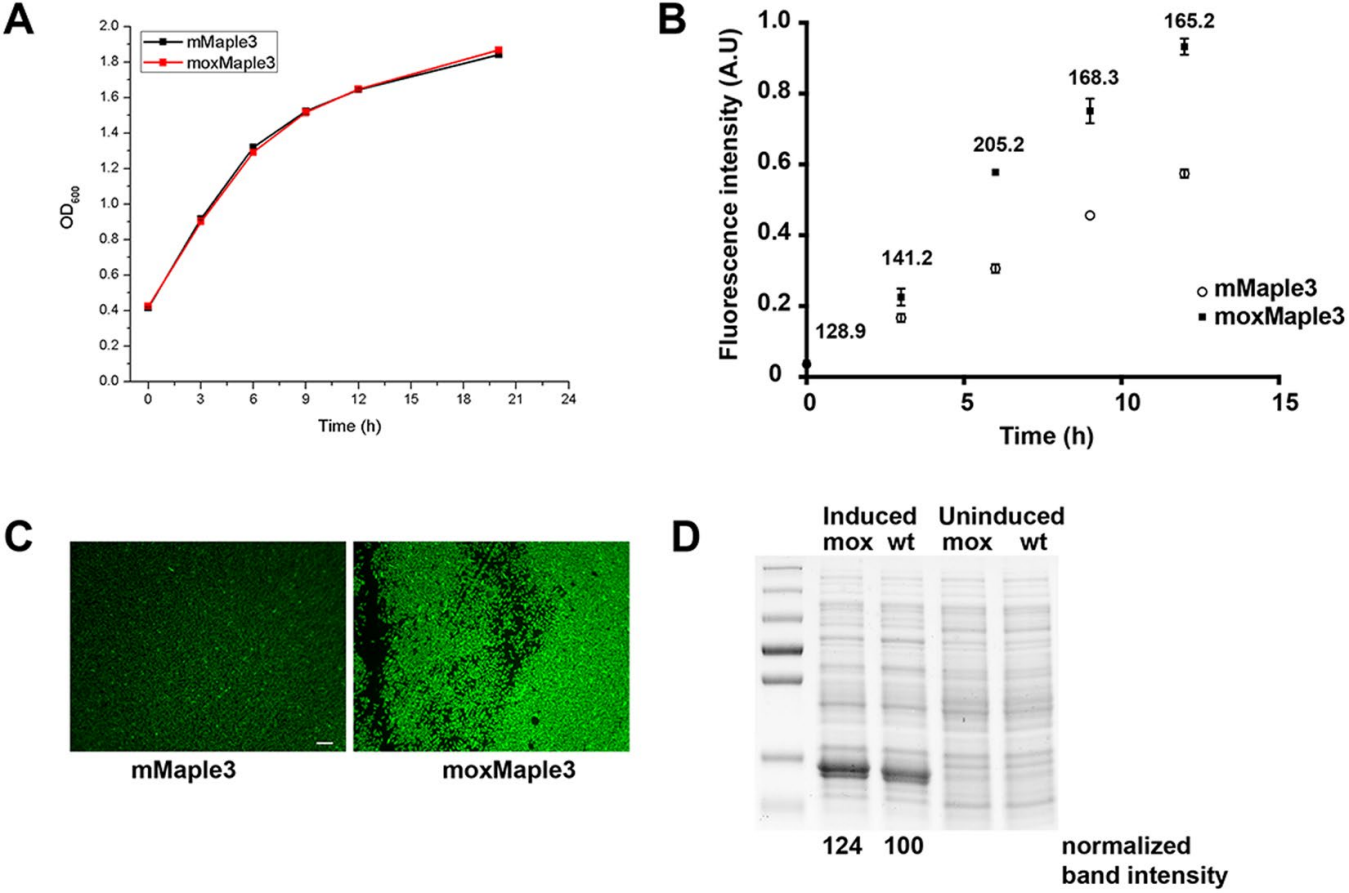
Bacterial expression of mMaple3 and moxMaple3 reveals significant differences in bacterial brightness. (**A**) Growth curves of liquid cultures of bacteria expressing mMaple3 or moxMaple3 exhibit no difference in growth rates. (**B**) Brightness of induced bacterial cultures exhibit substantial differences at all time points analysed. Means +/− S.E.M. are shown for each time point for each bacterial strain. The ratio of moxMaple3 fluorescence intensity relative to the mean intensity of the wt mMaple3-expressing bacteria are reported above each time point. Mean differences were statistically significant (p < 0.01) for all times except times 0 and 3 h (using a two tailed t-test). (**C**) Widefield fluorescence micrograph of bacteria expressing mMaple3 or moxMaple3 after 24 h. Note the dramatic difference in fluorescence intensities. Scale bar = 10 µm. (**D**) Coomassie stained SDS-PAGE gel of lysates of bacterial strains expressing moxMaple3 (mox) or mMaple3 (wt) after no induction or 24 h of induction with arabinose (see Supplementary Fig. S5 for the original gel). The intense bands in the induced lanes were quantified using ImageJ and then normalized relative to the intensity of the mox band. moxMaple3 is expressed at levels ~25% higher than mMaple3.

Upon successfully adapting mMaple3 with mutations, we characterized the resulting FP and sought to determine how the mutations impacted bacterial brightness. We considered four possibilities: i) decreased toxicity of the FP resulting in improved cell growth, ii) improved translation and consequently higher protein levels, potentially through improved mRNA structure, iii) increased FP brightness, and/or iv) improved folding resulting in higher levels of correctly folded and thus chromophore-forming protein. We began by asking whether mutations at the Cys110 position improved bacterial growth, potentially as a consequence of decreased FP toxicity. In Fig. 3A, we observed no difference in growth rates for bacteria expressing the parent or mox variants. Therefore, we discarded the hypothesis that the FP might impact cell growth or viability.

Next, we asked whether individual bacteria expressing moxMaple3 were inherently brighter than bacteria expressing mMaple3. moxMaple3 expressing cells were clearly brighter (Fig. 3B,C). This result suggested that more FP might be expressed or the properties of the new FP increased bacterial brightness. To compare absolute protein levels, Coomassie-stained polyacrylamide gels of bacterial lysates were generated for bacterial strains induced and expressing the parental and mox variants. A small, but significant increase of ~25% in band intensity for the moxMaple3 variant argued that a part of the increased bacterial brightness was, indeed, due to enhanced protein expression (Fig. 3D). A similar observation was made by Duwe’ *et al*. when they evolved EGFP to the reversibly photoswitchable rsEGFP and rsEGFP2 and observed increased FP expression in bacteria^21^.

As we could map the enhanced expression to a single amino acid, we considered two potential explanations for increased protein levels– either the changes in the mRNA sequence might enhance translation or the cysteine mutation, itself, improved protein stability. While mRNA structures are notoriously difficult to predict using algorithms, nonetheless, we found that nucleotide changes for moxMaple3 could dramatically alter the predicted secondary structure relative to mMaple3 mRNA (Fig. S1). To further test if these structural differences could alter expression, we generated a series of mutations to alter codons and amino acids at Cys110 and then compared bacterial brightness (Table 1). Substituting the other cysteine codon (TGC to TGT) had no impact on brightness. This argued that the codon wobble position was unlikely to be critical to protein expression or brightness. A series of codons for different amino acids at position 110 had a range of effects on protein brightness, but no obvious correlation between a particular nucleotide at a position and bacterial brightness was observed. While our mutagenesis at this position was not exhaustive, a pattern emerged that suggested that hydrophobic amino acids (Ala, Ile, and especially Val) in position 110 generally improved bacterial brightness, while a hydrophilic Thr did not (Table 1). The benefit of the Val mutation was unique to Cys110. Mutation of Cys180 to Ala or Val decreased bacterial brightness (Table 1). Interestingly, the original *Clavularia*-derived FP, mTFP1, has a Val at the equivalent 110 codon position. This observation suggested that the process of evolving new FPs from mTFP1 had inadvertently adversely impacted one or more properties of the resulting FPs due to loss of an optimal amino acid.

**Figure 4:**
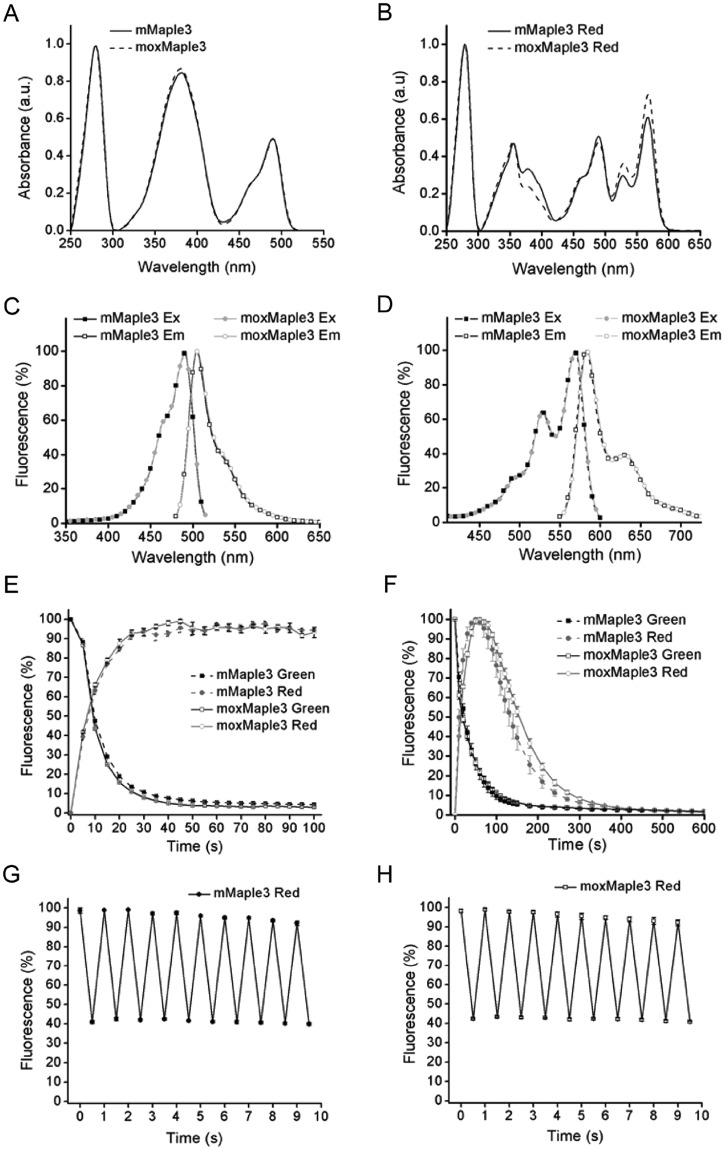
Spectral properties of moxMaple3. Absorbance spectra of mMaple3 and moxMaple3 in the green state (**A**) and after photoswitching (**B**). Excitation and emission spectra of mMaple3 and moxMaple3 in the green state (**C**) and after photoswitching (**D**). Kinetics of photoswitching by 390/40 nm light of mMaple3 and moxMaple3 (**E**). Kinetics of mMaple3 and moxMaple3 proteins in the photostability of the red state (**F**). Reversible photoswitching of photoconverted mMaple3 (**G**) and moxMaple3 (**H**) with 570/30 nm and 480/40 nm light. The error bars indicate S.E.M. from 4 different experiments.

If bacterial brightness could only be partially explained by improved protein expression, we reasoned that FP properties must account for the improved bacterial brightness. Here, we received a surprise. Purification and characterization of mMaple3 and moxMaple3 revealed that the proteins had nearly identical spectral characteristics for both the green and photoactivated red versions of the FPs (Fig. 4 and Supplementary Table S1). moxMaple3 was not brighter. Therefore, coupled with expression level data, we concluded that the Cys110Val mutant must significantly (accounting for >50% of the increased bacterial brightness for moxMaple3) improve the fraction of protein that successfully folds and produces a stable chromophore. Based on the crystal structure of mTFP1, we hypothesized that V105 in mTFP1 forms a functionally significant contact with the highly conserved Ile65, found in all *Clavularia* FPs (Supplementary Fig. S2)^19^. The original mutations to monomerize mMaple3 may have decreased the robustness of mMaple3 folding and our C110V moxMaple3 mutation may have inadvertently restored a hydrophobic interaction with Ile65. Other hydrophobic substitutions at C110 support this interpretation (Table 1). Thus, the moxMaple3 C110V mutation could potentially improve the chromophore folding environment and the overall stability of the protein, accounting for our observations of improved expression and chromophore production. Our fortuitous finding raises the possibility that other *Clavularia* FPs (e.g. mClavGRs, Kaede, mKikGR) may benefit from the C110V mutation, as well. The outcome of our characterizations was striking in how much a single amino acid change could improve FP folding.

### Impact of Cys110 on mMaple3 maturation

We hypothesized that the C110V mutation improved either the fraction of proteins capable of chromophore formation or the rate of chromophore maturation. Wang *et al*. observed a two-fold higher fraction of mMaple vs. mMaple3 that could be imaged^13^. This was in part due to the ability of roughly 60% of mMaple molecules to undergo maturation and form chromophores, while mMaple3 matured less efficiently^13^. The authors also suggested that other PSFPs suffer more severely from issues of incomplete maturation and efficiency of photoconversion.

**Figure 5:**
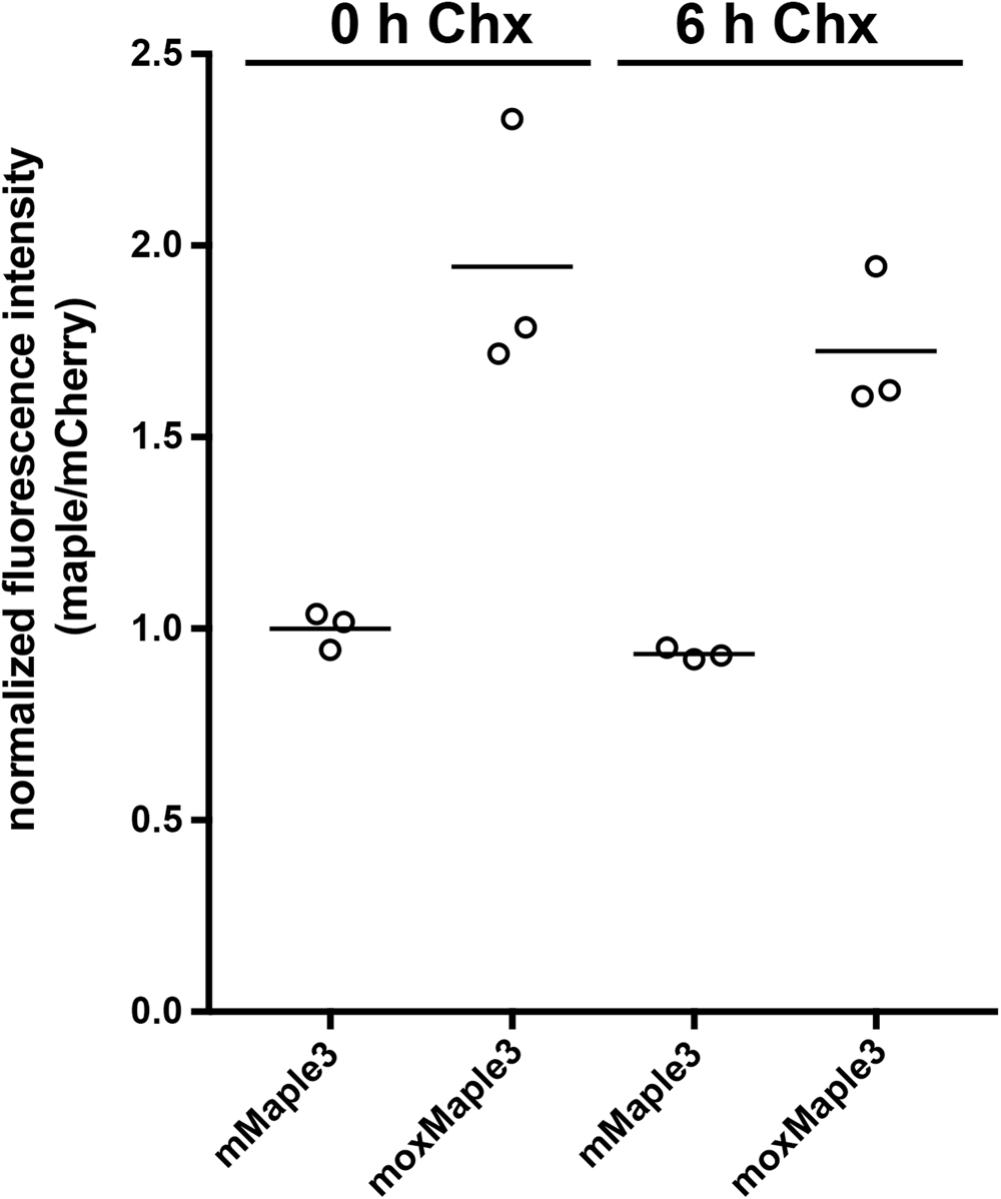
The difference in fluorescence intensities of cells expressing mCherry-T2A-mMaple3 vs. mCherry-T2A-moxMaple3 does not appear to be due to rates of chromophore maturation. Fluorescence intensities of cells were measured after treatment with cycloheximide (Chx) at 0 and 6 h and ratios of mMaple3 or moxMaple3 to the mCherry were determined and all values were normalized to 0 h Chx for mMaple/mCherry. No changes in fluorescence over time were observed (t-test, 2 tailed, p = 0.089). Interestingly, lysates of cells expressing moxMaple3 were nearly twice as bright as for cells expressing mMaple3. No significant difference in mCherry fluorescence over time was observed for either reporter (p > 0.1 mCherry-T2A-mMaple3, p > 0.5 mCherry-T2A-moxMaple3, maximum observed change in mCherry intensity >3%).

We investigated whether the apparent difference in mMaple3 and moxMaple3 maturation was due to differences in the rate of maturation or in the completeness of maturation. HeLa cells expressing cytoplasmic mMaple3 or moxMaple3 were treated with cycloheximide for 0 or 6 h to inhibit new protein synthesis. We predicted that if mMaple3 matures more slowly than moxMaple3 that there could be a significant increase in cellular fluorescence over time for mMaple3 expressing cells as pre-existing protein matures and forms chromophores. However, we did not observe any increase in mMaple3 fluorescence intensity in cells after 6 h of translation inhibition, nor did moxMaple3 cell intensity increase, either (Fig. 5). We note that FP chromophore maturation half-times range typically from a few minutes to 2–3 h^22^. Therefore, 6 h should have been sufficient to observe any additional mMaple3 maturation and concomitant increase in cell brightness, if maturation was simply slower. The absence of evidence of delayed maturation of a substantial population of mMaple3 molecules suggests that during folding, nascent mMaple3 molecules could become trapped in a terminal nonfluorescent apoprotein conformation. Moreover, similar to bacterial cells, we observed an almost 2-fold higher cellular fluorescence for moxMaple3 expressing HeLa cells. In contrast, the overall level of protein expression for both variants, as monitored by the fluorescence signal from mCherry, was the same. There is precedent for incomplete maturation of FPs. Chromophores of some FPs readily form more than one chemical structure^23^ and up to 30% of the common GFP fails to undergo the final dehydration reaction to form a fluorescent chromophore and remains in a terminal apoprotein form^24^. Bacteria expressing Venus FP are dramatically brighter than those expressing the parental EYFP despite similar brightness of the purified FPs^7^. Our data are consistent with a model in which the moxMaple3 mutations increase the probability of complete conversion of the apoprotein to a mature chromophore-containing protein.

### moxMaple3 performance in mammalian cells

Importantly, we wanted to establish that moxMaple3 performed at least as well as mMaple3 in mammalian cells as a fusion protein and for optical highlighting applications. First, we tested whether our mutations had impacted the monomericity of the FP. We fused the parent mMaple3 and moxMaple3 variant separately to the CytERM construct^21^ to assess whether either version induced formation of stacked ER membrane whorls, termed OSER. The monomericity of mMaple3 had been previously assessed in a bacterial protein clumping assay^13^. We observed 1 cell with OSER (n = 100 cells) for mMaple3 and 3 cells with OSER (n = 90) for moxMaple3 (Supplementary Fig. S3). Overall, these represent negligible levels of FP oligomerization and we classify both variants as monomeric. It is also worth noting that cells expressing the CytERM constructs exhibited qualitatively substantial differences in brightness, similar to what we observed for bacteria and for cytoplasmic expression in HeLa cells. Cells expressing CytERM-moxMaple3 were systematically brighter A similar difference in intensities was observed for cells expressing constructs, GalT-mMaple3 and GalT-moxMaple3, in which the FP was localized in the lumen of the Golgi complex, an oxidizing environment (Supplementary Fig. S4). Taken together, moxMaple3 label cells and cell structures more intensely than the parental mMaple3.

We also asked whether moxMaple3 impaired localization of a Golgi complex reporter. Costantini *et al*.^5^ and Kaberniuk *et al*.^9^ previously reported mislocalization of Golgi-targeted coral-derived red FPs in puncta within and distinct from the Golgi complex. We found that a Golgi complex targeted moxMaple3 reporter robustly co-localized with an endogenous Golgi complex marker, GM130 (Supplementary Fig. S5). Thus, moxMaple3 appears to be an inert reporter in cells and is suitable for use in fusion proteins.

**Figure 6:**
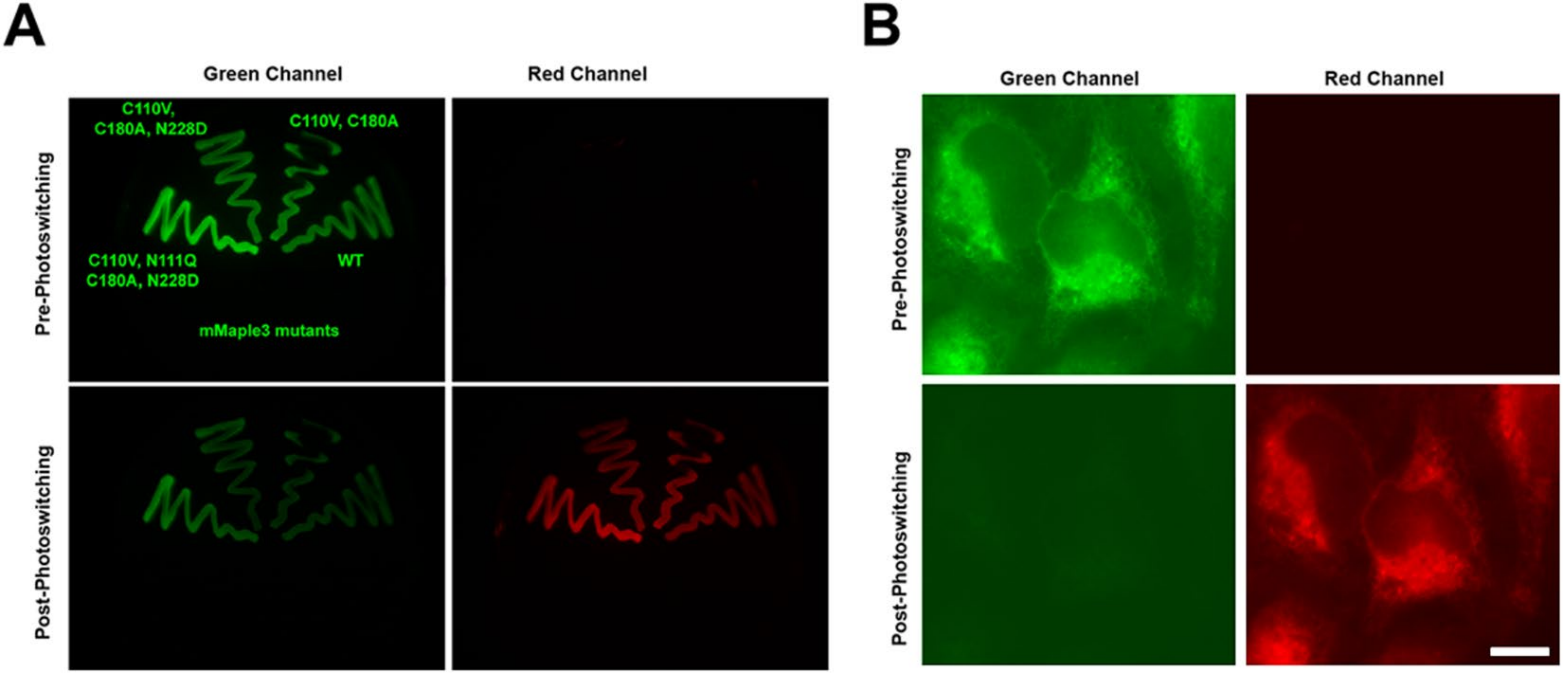
moxMaple3 maintains photoswitching properties in bacteria (**A**) and in mammalian cell ER (**B**). (**A**) Bacteria expressing the indicated mutants of mMaple3 were induced to express the FPs and then imaged and photoconverted using a 405/20 nm LED array, as described in the Experimental Section. All of the variants convert and the moxMaple3 bacteria are noticeably brighter for both pre-converted green and converted red states. (**B**) HeLa cells expressing ER-moxMaple3 were photoconverted on a widefield scope and the FP exhibits robust photo-conversion. Scale bar, 10 µm.

**Figure 7:**
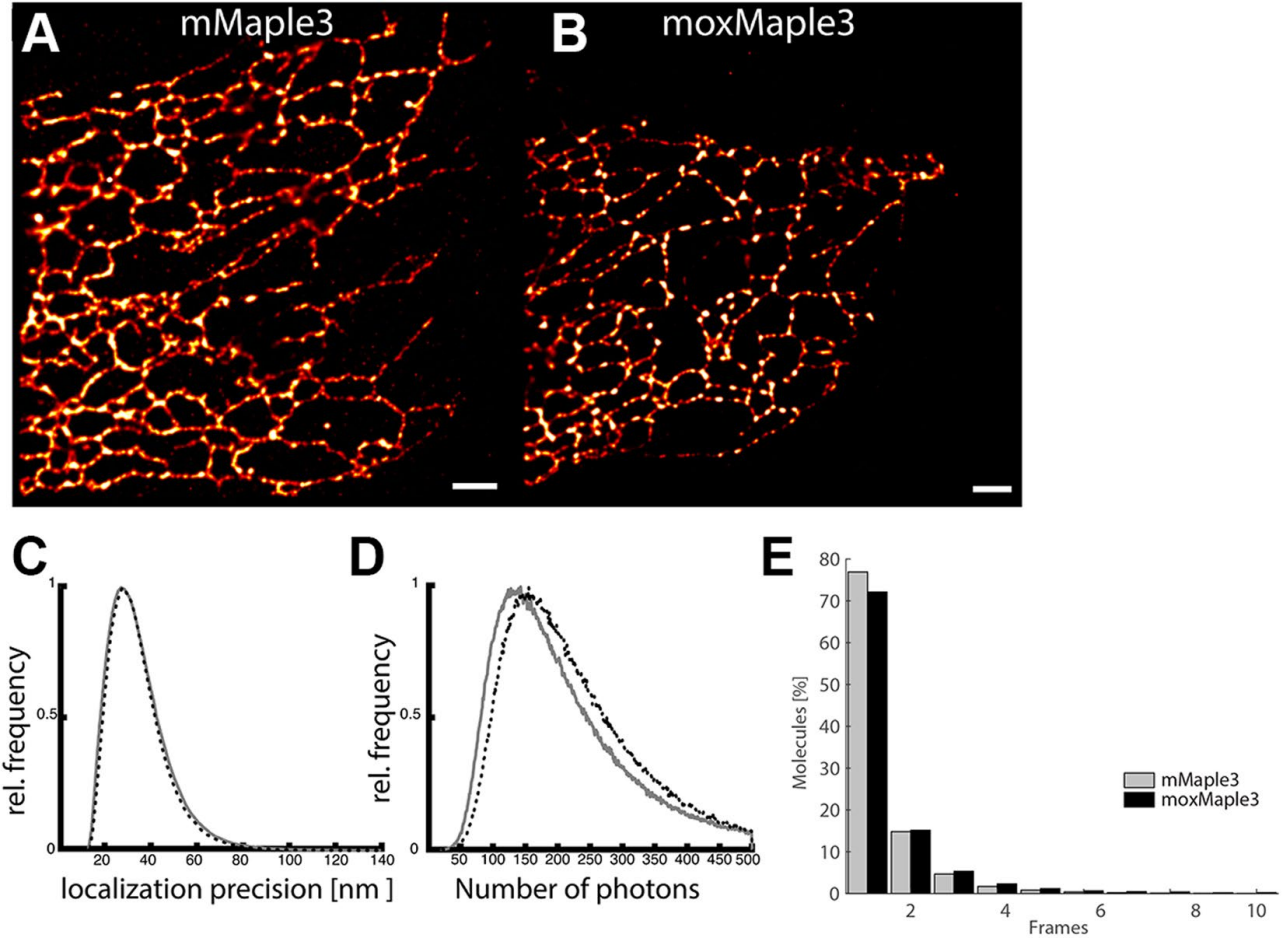
moxMaple3 excels for PALM microscopy of the ER. Distribution of localization precision for each molecule in cells expressing ER-mMaple3 (**A**) and ER-moxMaple3 (**B**). (**C** and **D**) Distributions of the localization precision and number of photons collected per molecule in cells expressing ER-mMaple3 and ER-moxMaple3. (**E**) Distribution of duration of molecular fluorescence (0.05 s frames) in cells expressing ER-mMaple3 (mean, 1.16; median, 1) and ER-moxMaple3 (mean 1.82, median, 1). Scale bar, 1 µm.

Finally, we confirmed that we could photoswitch the new moxMaple3 variant inside the oxidizing environment of the ER (Fig. 6) and thus moxMaple3’s suitability for PALM super-resolution imaging. As the photochemical properties of mMaple3 and moxMaple3 were nearly identical, we did not anticipate any gross differences in performance of the FPs. mMaple3 or moxMaple3 were separately targeted to the ER lumen in HeLa cells. Both constructs were readily photoswitched and usable in PALM assays, though moxMaple3 performed slightly better (Fig. 7). Specifically, for PALM, we observed a 5.5% increase in performance for localization precision means for ER-moxMaple3 relative to ER-mMaple3 (43.1 nm vs. 45.6 nm) (Fig. 7C) and a 29.8% higher mean photon count for ER-moxMaple3 relative to ER-mMaple3 (202.8 vs. 156.3) (Fig. 7D).

## Discussion

Attempts to study protein behaviors in living cells depend heavily on the inertness of reporter FPs fused to the endogenous proteins. Furthermore, the properties of FPs including brightness, rate of chromophore maturation, and photostability are top considerations. However, completeness of chromophore maturation is not typically measured in FP development studies. That is, the presence of a fluorescent signal is of primary importance while the number of fluorescent molecules is less critical. Yet, the completeness of FP maturation is especially important for quantitative studies in which investigators assume the number of fluorescent molecules equals the number of molecules tagged with the FP.

Previous studies revealed that a fraction of the FPs can misfold in some cellular environments and not produce chromophores while the rest of the population folds and fluoresces^3,5^. Our finding that C110V significantly increases the fraction of moxMaple3 that forms a chromophore highlights a second mechanism by which the number of fluorescent molecules may differ from the number of FP-tagged molecules. Thus, efforts to quantitatively study endogenous cellular proteins tagged with FPs using CRISPR approaches will benefit from validating or using validated FPs capable of correctly and maximally maturing into fluorescent molecules in the cellular environments to be studied. Alternatively, the incompleteness of FP maturation at least should be characterized and factored into any quantitative analyses, e.g. the population of visible fusion proteins is a fraction of the total population of tagged fusion proteins.

We sought to update mMaple3 for use in a variety of cellular environments through a set of targeted mutations. As such, our goal was not to create a brighter FP or one with new properties. Rather, we simply sought to improve the correct folding and utility of mMaple3 in oxidizing and secretory environments. Our improved moxMaple3 met these goals and unexpectedly increased successful chromophore maturation. The improved protein should be suitable for use in a wide variety of studies both for PALM microscopy and fusion protein fate studies. moxMaple3 now appears to be the best performing monomeric photoswitchable FP for use in PALM approaches.

## Materials and Methods

### Plasmid constructs

See Supplemental Data and Figures.

### Spectral and photochemical characterization of mMaple3 and moxMaple3

For bacterial expression of the mMaple3 and moxMaple3 nucleotide sequences encoding these proteins were subcloned into a pBAD/His-D vector using *Bgl*II and *Eco*RI restriction sites. Proteins with 6x polyhistidine tags on the N-terminus were expressed in BL21-AI bacteria. The bacteria were grown in RM medium supplemented with ampicillin for 6–8 h followed by an induction of the protein expression by adding of 0.1% arabinose. The proteins were purified using a Ni-NTA agarose (Qiagen, Germantown, MD, USA).

For absorbance measurements, a Hitachi U-2000 spectrophotometer was used. Fluorescence was measured by a FluoroMax-3 spectrofluorometer (Horiba-Jobin Yvon, Edison, NJ, USA). Photoconversion of purified proteins was performed with a 405/20 nm custom assembled LED array. Proteins were diluted with PBS pH 7.5 to 5 µM and placed on ice in transparent micro tubes with following illumination at 15 mW cm^−2^ for 20 min.

To determine molar extinction coefficients of the green chromophores, we measured mature chromophore concentrations. Purified proteins were alkali-denatured and extinction coefficients was calculated according to the known extinction coefficient (44,000 M^−1^ cm^−1^) of the synthetic compound of the tyrosine-containing GFP-like chromophores at 447 nm in 1 M NaOH^25^. Based on the absorbance of the native and denatured proteins, molar extinction coefficients for the native states were calculated. Molar extinction coefficients of the red chromophores were estimated by comparing the absorption bands corresponding to the pre-photoconverted green species and the absorption band of the red species from photoconverted samples.

Fluorescence quantum yield of the green and red chromophores of mMaple3 and moxMaple3 were measured relative to EGFP and TagRFP^26^ respectively, according to Williams’s method^27^.

To evaluate photostability, photoconversion kinetics and reversible photoswitching of the red form, the BL21-AI bacterial cells containing target proteins expressed from a pBAD/His-D plasmid were grown on LB/ampicillin Petri dishes supplemented with 0.1% arabinose, overnight at 37 °C. Bacteria were photobleached using an Olympus IX81 inverted epifluorescence microscope equipped with a 300 W Xenon arc lamp (LB-LS/30, Sutter), 60 × 1.35 NA oil-immersion objective lens (UPlanSApo, Olympus) and 480/40 nm exciter (Olympus Corporation, Center Valley, PA USA). Since 570/30 nm light caused reversible photoconversion of the chromophore from the red to green state, a 480/40 nm exciter was used for measurement of photostability of proteins before and after photoconversion. The intensity of the photobleaching light was measured as 14.7 mW cm^−2^ at the back focal plane of objective lens. Fluorescence of the green and red forms was detected using a 480/40 nm exciter and 535/40 nm emission filter and a 570/30 nm exciter and 615/30 nm emission filter sets respectively (Chroma Technology Corporation, Bellows Falls, VT). For measurement of the photoconversion halftime, a 390/40 nm exciter was used. The intensity of the photoconversion light was 1.3 mW cm^−2^ when measured at the back focal plane of a 60 × 1.35 NA oil-immersion objective lens. After photoconversion with 390/40 nm light, as described above, reversible photoswitching of proteins in the red state was performed using 570/30 nm and 480/40 nm light with intensities 32 mW cm^−2^ and 27 mW cm^−2^ respectively measured at the back focal plane of objective lens with exposure time 0.5 s.

To test whether mMaple3 matured more slowly than moxMaple3, mMaple or moxMaple3 was cloned into a mCherry-T2A-[maple variant] expression plasmid (derived from the Clontech N1-EGFP plasmid). HeLa cells grown in 12-well plates were transfected using Effectene (Qiagen) and then treated 36 h post-transfection with 50 mg/ml cycloheximide (Acros Organics, cat #357420010) for 0 or 6 h. Cells were trypsinized, collected by centrifugation at 1000 × g 5 min, washed with 1x PBS, and then lysed in M-PER solution (Mammalian Protein Extraction Reagent, Thermo Scientific cat #78501) and fluorescence intensities were measured (for mCherry using excitation 530 nm, emission 545–700 nm and for Maple3 variants excitation 455 nm and emission 475–600 nm) with a FluoroMax-3 spectrofluorometer (Horiba-Jobin Yvon Inc. Edison, NJ, USA). The green fluorescence intensities were divided by the respective mCherry fluorescence intensities to normalize for expression and then all values were normalized to the mean intensity of mMaple3 at 0 h cycloheximide.

### RNA structure prediction

RNA secondary structure was predicted using the Predict a Secondary Structure server on default settings for RNAstructure software^28^. The structures generated for each construct individually were highly similar and only the first predicted structure is shown for each prediction.

### Cell culture and transfection

HeLa cells were routinely cultured in RPMI medium (Mediatech, Manassas, VA), supplemented with 5 mM glutamine, penicillin/streptomycin (Invitrogen, Carlsbad, CA), and 10% heat inactivated fetal bovine serum (Hyclone from Thermo Scientific, Rockford, IL) at 37 °C in 5% CO_2_ for imaging experiments, cells were grown in 8-well LabTek coverglass chambers (Nunc, Rochester, NY). All constructs were transiently transfected for 16–48 h into cells using Lipofectamine 2000 (Invitrogen, of Thermofisher Scientific Waltham, MA) according to the manufacturer’s instructions.

### Live cell fluorescence imaging

Live cells were imaged in phenol red-free RPMI supplemented with 10 mM Hepes (Fisher Scientific, Waltham, MA USA) and 10% fetal bovine serum. Cells were imaged with an Axiovert 200 widefield fluorescence microscope (Carl Zeiss Microimaging, Thornwood, NY USA) with a 63x oil NA 1.4 objective, 450–490 excitation/500–550 emission bandpass filter for the green forms of mMaple3 and moxMaple3 and a 565/30 nm excitation-620/60 nm emission bandpass filter for the red forms of mMaple3 and moxMaple3. Photoconversion was performed by exciting cells for 30–45 s with a DAPI filter (365 nm ex and 445/50 nm emission). Composite figures were prepared using ImageJ (National Institutes of Health, Bethesda, MD USA), Photoshop CC 2017 and Illustrator CC 2017 software (Adobe Systems, San Jose, CA USA). Image analysis and composite figures were prepared using ImageJ, and Prism (GraphPad Software, La Jolla, CA USA).

### Immunofluorescence

HeLa cells grown in 8-well LabTek dishes were transfected with GalT-mMaple3 or GalT-moxMaple3 using Lipofectamine2000 as per manufacturer’s recommendations. 16 h post-transfection, cells were fixed in 4% paraformaldehyde in 1x PBS for 20 min. Cells were then permeabilized with 0.1% triton X-100 in 1x PBS. Cells were blocked in RPMI1640 containing 10% FBS for 30 min. Cells were incubated with anti-GM130 (anti-GM130, BD Transduction Laboratories catalogue number 610823, 1:500 BD Biosciences, San Jose, CA) followed by Alexa Fluor 555 conjugated anti-rabbit IgG secondary antibody (catalogue number A21428 used at 1:500, Life Technologies) in the RPMI block solution. Cells were imaged in 1x PBS on a ZEISS LSM880 AxioObserver with a EC Plan-Neofluar 40x oil immersion objective (NA:1.3) using 488 nm and 561 nm laser excitation through a MBS 488/561 nm dichroic and a BP495–550 + LP570 nm emission filter. z-stacks contained 34 slices (1136 × 1136 px) in 0.2 µm intervals and were subject to SR-Airyscan processing (ZEISS ZEN Black) and maximum intensity projection. Linear contrast enhancement was performed in Fiji software (http://fiji.sc/Fiji).

### Photoactivation localization microscopy

PALM experiments were carried out on a commercial Zeiss ELYRA TIRF-PALM-SIM microscope system (Carl Zeiss Microimaging), with a Plan-Apochromat 100 × 1.4 NA objective as reported previously^29^. Mycoplasma-free HELA cells were transfected with pER-moxMaple3 and pER-mMaple3 using Lipofectamine 2000 (Thermo Fisher) according to the manufacturer’s recommendations, incubated at 37 **°**C, 5% CO_2_ in the dark for 24 h before they were fixed in 4% PFA and prepared as described previously^30^. Fixed cells were imaged in 1x PBS at room temperature. Samples were converted at 405 nm and imaged at 561 nm using 50 ms frames and a BP420–480 + BP570–640 + LP740 nm emission filter as described before^30^. Images were drift corrected, reconstructed, and analysed using the ZEN Software and custom MATLAB scripts. After PALM reconstruction, images were minimally processed using the image analysis software Fiji. Specifically, linear contrast/brightness enhancements were applied to the images.

## Electronic supplementary material

Supplementary information

## Data Availability

The datasets generated and/or analysed during the current study are available from the corresponding authors on reasonable request.
