# Supplementary material for: moxMaple3: a Photoswitchable Fluorescent Protein for PALM and Protein Highlighting in Oxidizing Cellular Environments
Source: Sci Rep. 2018 Oct 3;8:14738. doi: 10.1038/s41598-018-32955-5 (PMC6170497; doi:10.1038/s41598-018-32955-5)
Supplement: Supplementary file 1 — Supplementary information [file 41598_2018_32955_MOESM1_ESM.pdf]

Supplementary Information and Figures for

moxMaple3: a Photoswitchable Fluorescent Protein for PALM and Protein Highlighting in Oxidizing Cellular Environments

Andrii A. Kaberniuk,[a] Manuel A. Mohr,[b] Vladislav V. Verkhusha,[a*] and Erik Lee Snapp[b*]

[a]Albert Einstein College of Medicine, Department of Anatomy and Structural Biology and the Gruss-Lipper Biophotonics Center, 1300 Morris Park Avenue, Bronx, NY 10461;

[b]Janelia Research Campus, HHMI, 19700 Helix Drive, Ashburn, VA 20147

*Address correspondence to: Erik Snapp at snappe@janelia.hhmi.org and Vladislav Verkhusha at vladislav.verkhusha@einstein.yu.edu

Supplementary Methods: Plasmid Constructs and Sequences

Engineering of codon optimized mMaple3.

mMaple3 was synthesized by GenScript (Piscataway, NJ) using the high expression human optimized codons described by Haas et al.[1]

mMaple3 coding sequence

ATGGTGAGCAAGGGCGAGGAGACCATCATGAGCGTGATCAAGCCCGACATGAAGATCAAGC
TGCGCATGGAGGGCAACGTGAACGGCCACGCCTTCGTGATCGAGGGCGAGGGCAGCGGCAA
GCCCTTCGAGGGCATCCAGACCATCGACCTGGAGGTGAAGGAGGGCGCCCCCCTGCCCTTCG
CCTACGACATCCTGACCACCGCCTTCCACTACGGCAACCGCGTGTTCACCAAGTACCCCCGC
AAGATCCCCGACTACTTCAAGCAGAGCTTCCCCGAGGGCTACAGCTGGGAGCGCAGCATGA
CCTACGAGGACGGCGGCATCTGCAACGCCACCAACGACATCACCATGGAGGAGGACAGCTT
CATCAACAAGATCCACTTCAAGGGCACCAACTTCCCCCCCAACGGCCCCGTGATGCAGAAGC
GCACCGTGGGCTGGGAGGTGAGCACCGAGAAGATGTACGTGCGCGACGGCGTGCTGAAGGG
CGACGTGAAGATGAAGCTGCTGCTGAAGGGCGGCAGCCACTACCGCTGCGACTTCCGCACC
ACCTACAAGGTGAAGCAGAAGGCCGTGAAGCTGCCCAAGGCCCACTTCGTGGACCACCGCA
TCGAGATCCTGAGCCACGACAAGGACTACAACAAGGTGAAGCTGTACGAGCACGCCGTGGC
CCGCAACAGCACCGACAGCATGGACGAGCTGTACAAGTAA

mMaple3 protein

MVSKGEETIMSVIKPDMKIKLRMEGNVNGHAFVIEGEGSGKPFEGIQTIDLEVKEGAPLPFAYDIL
TTAFHYGNRVFTKYPRKIPDYFKQSFPEGYSWERSMTYEDGGICNATNDITMEEDSFINKIHFKG
TNFPPNGPVMQKRTVGWEVSTEKMYVRDGVLKGDVKMKLLLKGGSHYRCDFRTTYKVKQKA
VKLPKAHFVDHRIEILSHDKDYNKVKLYEHAVARNSTDSMDELYK*

Engineering moxMaple3.

The mMaple3 sequence was inserted in-frame into the pBAD/His-D vector. moxMaple3 was engineered by replacing Cysteines 110 and 180 with Alanines or Valines, and the consensus N-linked Asparagines with Glutamine and Aspartic acid. Forward and reverse primers included:

mMaple3C110A/V F

CTACGAGGACGGCGGCATCGYCAACGCCACCAACGACATCACC

mMaple3C110A/V R

GGTGATGTCGTTGGTGGCGTTGRCGATGCCGCCGTCCTCGTAG

mMaple3C180A/V F

AGGGCGGCAGCCACTACCGCGYCGACTTCCGCACCACCTACAAGGTG

mMaple3C180A/V_R

CACCTTGTAGGTGGTGCGGAAGTCGRCGCGGTAGTGGCTGCCGCCCT

mMaple3N111Q F

GAGGACGGCGGCATCGTCCAGGCCACCAACGACATCACCATG

mMaple3N111Q R

CATGGTGATGTCGTTGGTGGCCTGGACGATGCCGCCGTCCTC

mMaple3N227D F

ACGCCGTGGCCCGCGACAGCACCGACAGCATGGACG

mMaple3N227D R

CGTCCATGCTGTCGGTGCTGTCGCGGGCCACGGCGT

These primers were then used to make single mutations and then the sugarless and cysteine-less moxMaple3. Sites of mutagenesis are underlined.

moxMaple3 coding sequence

ATGGTGAGCAAGGGCGAGGAGACCATCATGAGCGTGATCAAGCCCGACATGAAGATCAAGC
TGCGCATGGAGGGCAACGTGAACGGCCACGCCTTCGTGATCGAGGGCGAGGGCAGCGGCAA
GCCCTTCGAGGGCATCCAGACCATCGACCTGGAGGTGAAGGAGGGCGCCCCCCTGCCCTTCG
CCTACGACATCCTGACCACCGCCTTCCACTACGGCAACCGCGTGTTCACCAAGTACCCCCGC
AAGATCCCCGACTACTTCAAGCAGAGCTTCCCCGAGGGCTACAGCTGGGAGCGCAGCATGA
CCTACGAGGACGGCGGCATCGTCCAGGCCACCAACGACATCACCATGGAGGAGGACAGCTT
CATCAACAAGATCCACTTCAAGGGCACCAACTTCCCCCCCAACGGCCCCGTGATGCAGAAGC
GCACCGTGGGCTGGGAGGTGAGCACCGAGAAGATGTACGTGCGCGACGGCGTGCTGAAGGG
CGACGTGAAGATGAAGCTGCTGCTGAAGGGCGGCAGCCACTACCGCGCCGACTTCCGCACC
ACCTACAAGGTGAAGCAGAAGGCCGTGAAGCTGCCCAAGGCCCACTTCGTGGACCACCGCA
TCGAGATCCTGAGCCACGACAAGGACTACAACAAGGTGAAGCTGTACGAGCACGCCGTGGC
CCGCGACAGCACCGACAGCATGGACGAGCTGTACAAGTAA

moxMaple3 Protein

MVSKGEETIMSVIKPDMKIKLRMEGNVNGHAFVIEGEGSGKPFEGIQTIDLEVKEGAPLPFAYDIL
TTAFHYGNRVFTKYPRKIPDYFKQSFPEGYSWERSMTYEDGGIVQATNDITMEEDSFINKIHFKG
TNFPPNGPVMQKRTVGWEVSTEKMYVRDGVLKGDVKMKLLLKGGSHYRADFRTTYKVKQKA
VKLPKAHFVDHRIEILSHDKDYNKVKLYEHAVARDSTDSMDELYK*

To create ER-Maple3 and ER-moxMaple3, the coding sequences were PCR amplified and inserted into the *AgeI*/*NotI* sites of Clontech N1 vector containing an in-frame bovine prolactin signal sequence 5' to the fluorescent protein coding sequence using the following primers:

Forward mMaple3 *AgeI*

GGACCGGTCGCCACCATGGTGAGCAAGGGCGAGG

Reverse mMaple3 KDEL *NotI*

TCGCGGCCGCTTACAATTCATCCTTCTTGTACAGCTCGTCCATGCTG

Note that the reverse primer appends a KDEL ER retrieval motif at the end of the constructs.

To create CytERM-mMaple3 and CytERM-moxMaple3, constructs were cut at *AgeI*/*Not*I and the FP fragments were inserted into the *Age*I/*Not*I sites of CytERM-EGFP, replacing the EGFP fluorescent protein coding sequence with the mMaple3 variants.

Forward mMaple3 *AgeI*

GGACCGGTCGCCACCATGGTGAGCAAGGGCGAGG

Reverse mMaple3 *NotI*

TCGCGGCCGCTTACTTGTACAGCTCGTCCATGCTG

REFERENCES

1. Haas, J., Park, E. C. & Seed, B. Codon usage limitation in the expression of HIV-1 envelope glycoprotein. *Curr Biol* **6**, 315-324 (1996).

2. Ai, H. W., Olenych, S. G., Wong, P., Davidson, M. W. & Campbell, R. E. Hue-shifted monomeric variants of Clavularia cyan fluorescent protein: identification of the molecular determinants of color and applications in fluorescence imaging. *BMC Biol* **6**, 13(2008).

Supplementary Figure S1. Diagrams of predicted secondary structures of the mRNAs for mMaple3 and moxMaple3.

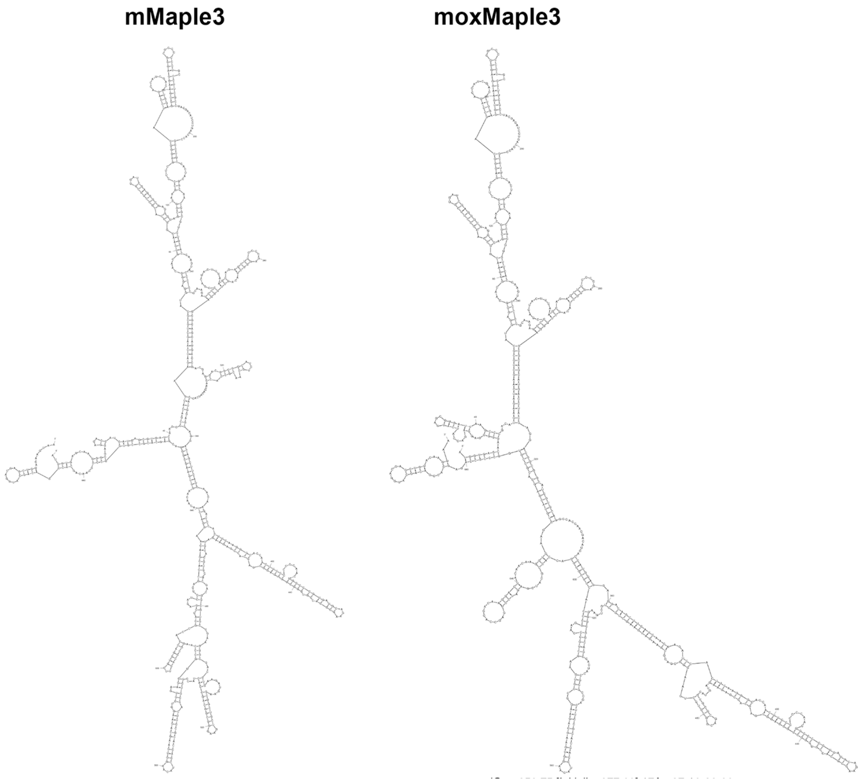

Supplementary Figure S2. Alignment of sequences of mMaple3, moxMaple3, and mTFP1, and structure of mTFP1. Position 65 (60 in mTFP1) and 110 (105 in mTFP1) are indicated with red and orange bars respectively. Below are two views of the 3D protein structure of mTFP1 (PDB:2hqk)[2] are displayed with the red Isoleucine and orange Valine defined.

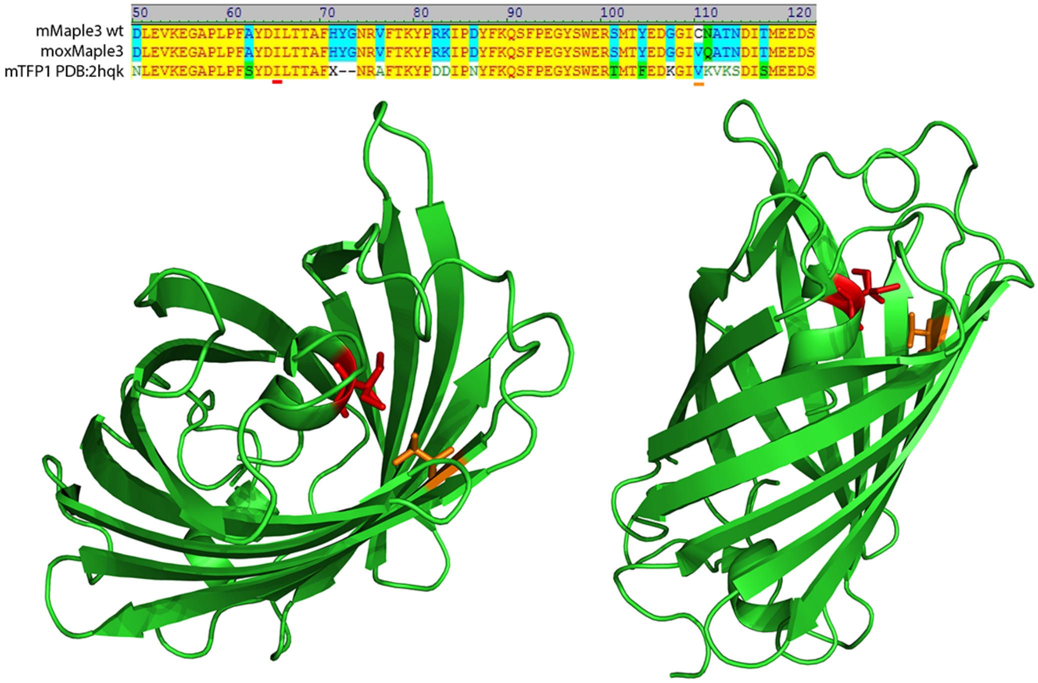

Supplementary Figure S3. Expression of CytERM-mMaple3 and CytERM-moxMaple3 in HeLa cells. The different fields of cells used for analysis of OSER structures are presented. Images are widefield fluorescence images in inverted grayscale for ease of visualization. Cells with OSER structures are indicated with an *. Note that CytERM-moxMaple3 cells are consistently brighter than cells expressing CytERM-mMaple3. Scale bar = 10 µm.

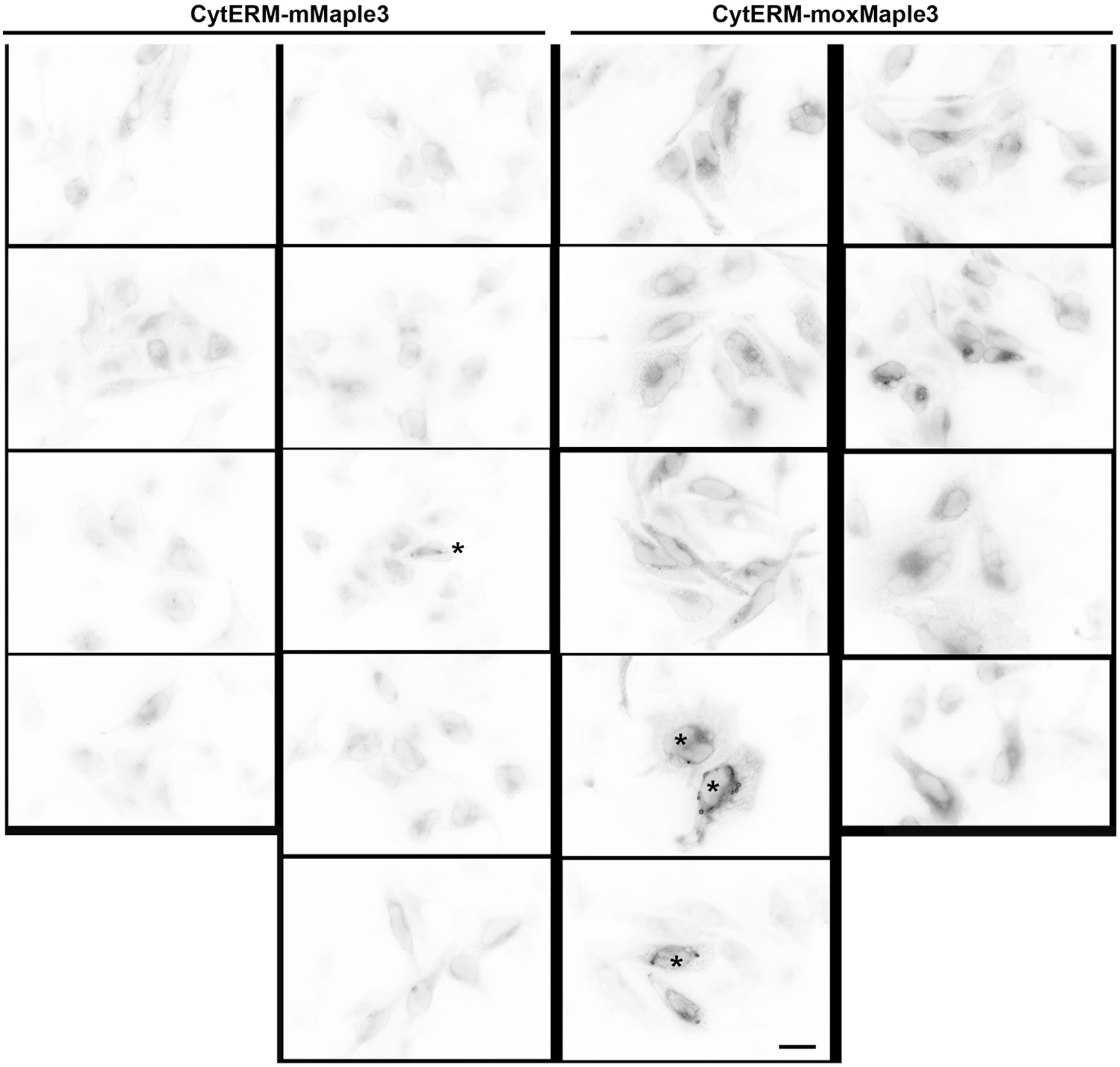

Supplementary Figure S4. Expression of GalT-mMaple3 and GalT-moxMaple3 in HeLa cells. Multiple fields of cells are presented. Images are widefield fluorescence images in inverted grayscale for ease of visualization. Dark perinuclear intensities correspond to Golgi complexes. Note that GalT-moxMaple3 cells are frequently more intense than cells expressing GalT-mMaple3. Scale bar = 10 µm.

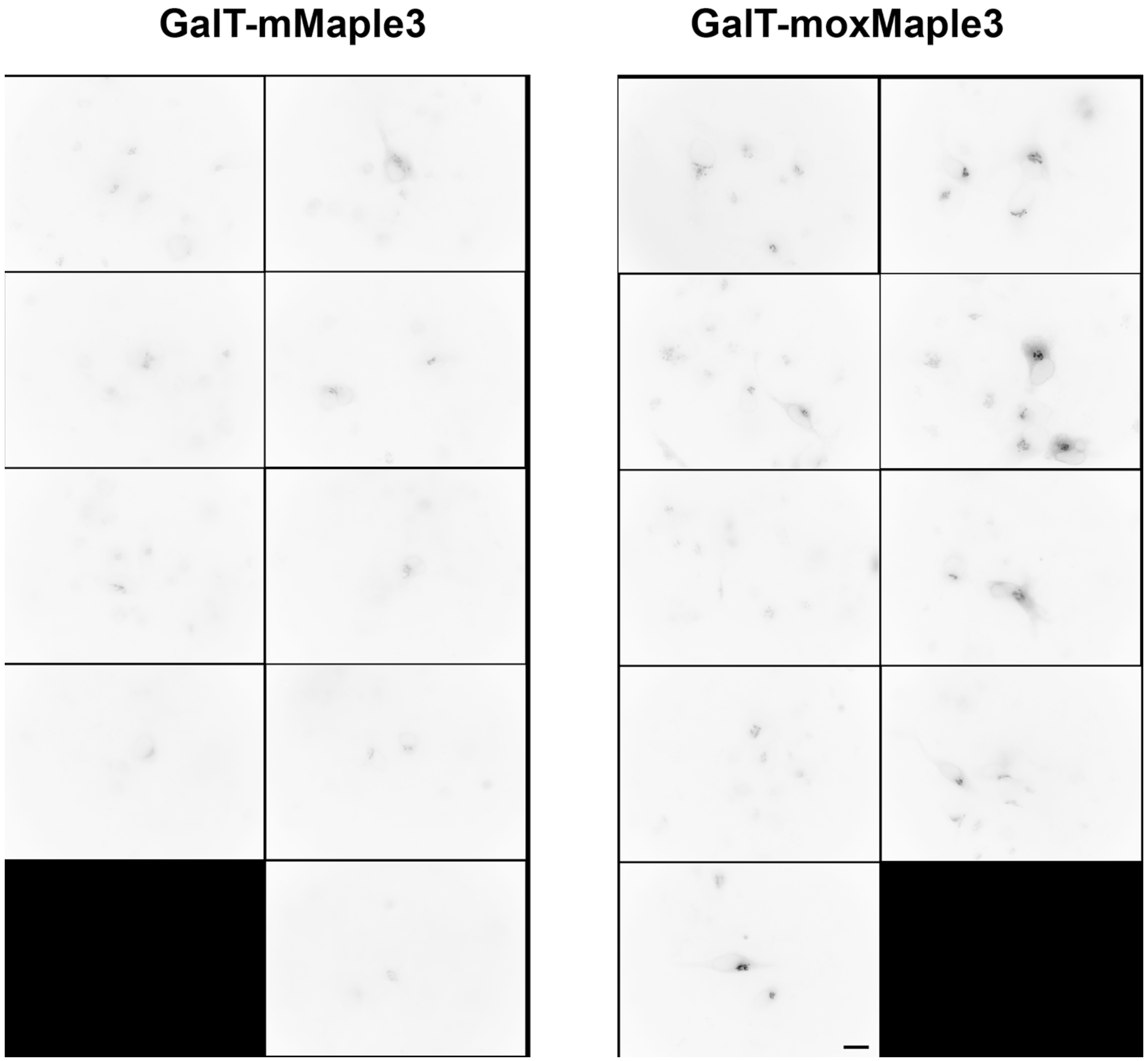

Supplementary Figure S5. Correct Golgi complex localization of GalT-moxMaple3 expressed in multiple HeLa cells stained with an antibody for the Golgi complex protein GM130. Images were acquired on a ZEISS LSM 880 AxioObserver with a EC Plan-Neofluar 40x oil immersion objective (NA:1.3) using 488 nm and 561 nm laser excitation through a MBS 488/561 dichroic and a BP495-550+LP570 emission filter. z-stacks contained 34 slices (1136x1136 px) in 0.2 µm intervals and were subject to SR-Airyscan processing (ZEISS ZEN Black) and maximum intensity projection. Linear contrast enhancement was performed in Fiji. Scale bar = 5 µm.

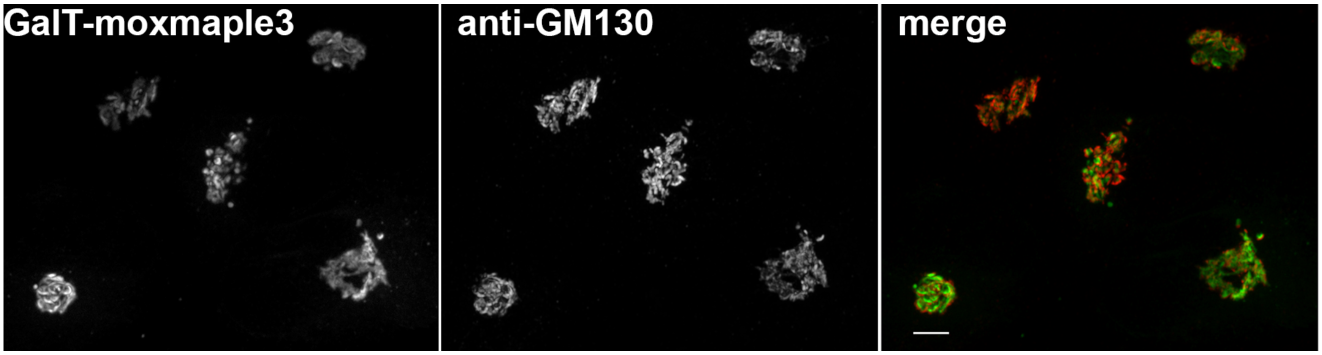

Supplementary Figure S6. Original Coomassie-stained gel for Figure 3D in the main text. Left four lanes are loaded with 5 µl of bacterial lysates and right four lanes are loaded with 10 µl of bacterial lysates. MW is Prestained Protein Ladder Thermoscientific #26619.

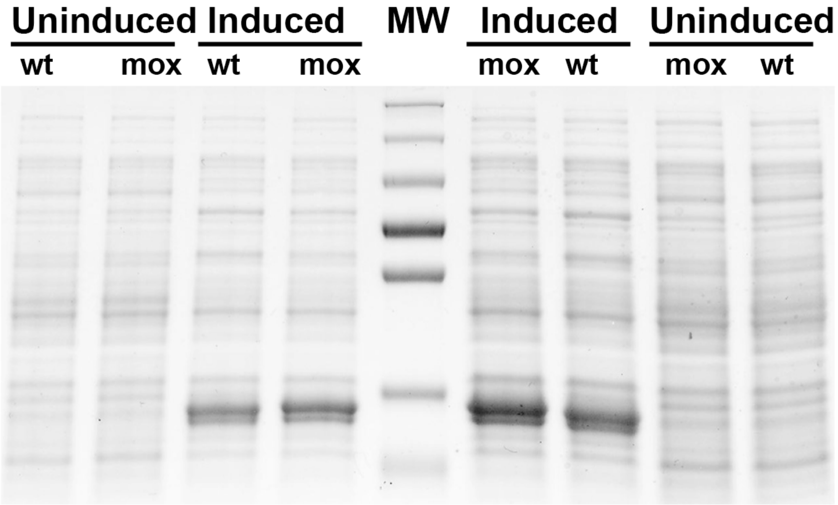

Supplementary Table S1. Spectral Characterization of mMaple3 and moxMaple3.

Parameter	mMaple3		moxMaple3	
	Green	Red	Green	Red
Excitation maximum (nm)	490	569	490	569
Emission maximum (nm)	505	582	506	584
Quantum yield	0.37	0.52	0.37	0.52
Extinction coefficient at pH 7.5 (M^{-1} cm^{-1})	15,760	23,970	14,800	24,230
Half-time of photostability (s)[a]	23	83	19	95
Half-time of photoconversion (s)	9		9	
Contrast of photoconversion, fold	>1,000		>1,000	
Molecular brightness[b]	0.17	0.37	0.16	0.38

[a] For photobleaching of chromophore in the red state, 480/40 nm light was used, since 570/30 nm light caused reversible photoconversion of the chromophore from the red to the green state.
[b] Brightness relative to EGFP taking extinction coefficient of 56,000 M^{-1} cm^{-1} and quantum yield of 0.6.
